# Gender differences in serum leptin concentrations from umbilical cord blood of newborn infants born to nondiabetic, gestational diabetic and type-2 diabetic mothers

**DOI:** 10.4103/0973-3930.57346

**Published:** 2009

**Authors:** Samsad Jahan, Rahelee Zinnat, Zahid Hassan, Kazal Boron Biswas, Samira Humaira Habib

**Affiliations:** Department of Gynecology and Obstetrics, Bangladesh Institute of Research and Rehabilitation in Diabetes Endocrine and Metabolic Disorders (BIRDEM), Dhaka, Bangladesh; 1 Biomedical Research Group (BMRG), BIRDEM, Dhaka, Bangladesh; 2 Health Economics Unit, Diabetic Association of Bangladesh (DAB), Dhaka, Bangladesh

**Keywords:** Serum leptin, Umbilical cord blood, Newborn infants, non diabetic, Type 2 Diabetic, gestational diabetes mellitus

## Abstract

To investigate gender differences, if any, in leptin concentrations from umbilical cord blood of new born infants of mothers with type 2 diabetes mellitus (DM), gestational diabetes mellitus (GDM), and Non diabetic (ND) at delivery. Serum leptin concentrations were measured in 105 newborns (53 males and 52 females in the three groups). Blood was taken from the umbilical cord of the babies at delivery. Maternal anthropometric measurements were recorded within 48 hours after delivery. Pearson correlation coefficient was used to explore the relationship between serum leptin concentrations and anthropometric measures of the fetus and their mother. Both Serum leptin level and serum C-peptide was measured by chemiluminescence based ELISA. The median range of leptin concentration in cord blood was ND group: Male [13.91 (3.22 – 47.63)], Female [16.88 (2 – 43.65)]; GDM group: Male [32 (7 – 76.00)], Female [36.73 (4.80 – 81.20)]; DM group: Male [20.90 (2 –76.00)], Female [32 {2.58 – 80.67)]. Cord serum leptin levels correlated with birth weight(r=0.587, p=0.0001), ponderal index (PI) (r=.319, p=0.024)of the babies and body mass index (BMI) (r=−0.299, p=0.035) of their mothers but did not correlate with gestational age, cord serum C-peptide concentration or placental weight at delivery. Leptin concentrations were higher in the female fetus in comparison to the male fetus. Birth weight of the female fetuses were also higher than that of male fetus. We found that there are very strong associations between cord leptin concentrations at delivery and birth weight, ponderal index of the baby, body mass index of the mothers with Type 2 DM. We also found that high leptin levels could represent an important feedback modulator of substrate supply and subsequently for adipose tissue status during late gestation or adipose tissue is the major determinant of circulating leptin levels.

## Introduction

The hormone, leptin, is an adipocyte secreting signal that contributes to the regulation of energy balance by informing the brain of the amounts of adipose tissue in the body, thereby regulating food intake and energy expenditure.[1–4]

In women, both the level of obmRNA in adipose tissues and the plasma leptin concentrations are higher than those in men and this has been attributed to relatively greater body fat contents or reproductive hormones.[5–7]

In a cross-sectional study of a large population of children of both sexes, it was observed that, at any age and at any pubertal stage studied, the girls always had higher leptin concentration than the boys.[8] This finding was evident even in the youngest age group studied, 5 years, which is a period of child development without any sex-related hormonal changes. These differences in serum leptin concentrations between boys and girls could not be explained by differences in weight, height, age or adiposity. It has been reported that leptin concentration is higher in cord blood of DM and GDM babies. Therefore, it is worthy to examine if gender difference exists in different leptinemic conditions.

In the present study, we measured leptin concentrations in umbilical cord serum of a large group of infants of both sexes born to mothers having type-2 DM, GDM, ND at the time of delivery.

## Materials and Methods

The study population consisted of 30 babies, 15 male and 15 female babies, from nondiabetic mothers (ND-babies. 30 babies, 17 male and 13 female babies, born to gestational diabetes mellitus mothers (GDM-babies), 45 babies from type-2 DM mothers (DM babies), of them 21 were male babies and 24 were female babies.

All of the newborns were healthy and their mothers had no remarkable illnesses during their pregnancy and none was taking any medication, except vitamins and iron supplements.

Age of all mothers: 25-35 years.

Gestational age at the time of delivery was calculated according to the LMP and confirmed by USG during first trimester.

Birth weights of the babies were measured using standard weighing balance. Placentas were delivered within 10 min after delivery of the infants. Placental weight was measured by a weighing balance.

A sample of venous cord blood was collected from each newborn just after delivery from placental side of umbilical cord.

The serum was immediately separated and frozen at - 70 C until analysis.

All of the parents of the newborns gave their written informed consent prior to enrollment.

Neonates born to mothers who experienced medical complications other than GDM and type-2 DM during, or before pregnancy were excluded.

Laboratory techniques: Serum leptin was measured by chemiluminescence-based ELISA (DPC, USA).

### Statistical analysis

Differences between groups were evaluated by Student's unpaired t-test. Significance was considered to be *P*<0.05. Data are presented as the median (range).

## Results

Birth weights of female babies of ND, GDM and type-2 DM groups were higher compared to the male babies of the respective groups [weight in kg, median (range) Table 1].

**Table 1:** Clinical and biochemical parameters of male and female fetuses of the study subjects

	Male (n = 15) median (range)	Female (n = 15) median (range)
	Nondiabetic	
Birth weight (kg)	2.646 (2 - 3.50)	2.72 (2.10 - 3.80)
Serum leptin concentration (ng/ml)	13.91 (3.22-47.63)	16.875 (2 - 43.65)
	GDM	
Birth weight (kg)	2.91 (2.10 - 4.50)	3.13 (2.20 - 4.50)
Serum leptin concentration (ng/ml)	32 (7 - 76.00)	36.73 (4.80-81.20)
	DM	
Birth weight (kg)	2.80 (2.30 - 3.60)	3.33 (1.70 - 4.80)
Serum leptin concentration (ng/ml)	20.90 (2 - 76.00)	32(2.58-80.67)

Serum leptin concentrations of female babies of ND, GDM and type-2 DM groups were also higher compared to the male babies of the same groups. [Leptin concentrations in (ng /ml), median (range)]

## Discussion

There is ample evidence providing differences in the leptin concentration between sexes.[12–14] Various mechanisms have been postulated to explain this difference. The most accepted explanation is the differential adiposity between the genders.[15–17] Others have proposed a heightened hypothalamic feedback loop in leptin adiposity regulation in the female.[18] The gender dimorphism in leptin production which is observed in the very early life may also indicate the genetic difference in leptin production.[19]

Higher leptin in the offspring of diabetic mother has been largely attributed to the increase in the adiposity of the offspring of diabetic mother. Others have proposed a regulatory role of insulin in the production of leptin. Placental production of leptin might be responsible for hyperinsulinemia in the offspring of DM mother.[20]

The mechanism of production and regulation of leptin concentration in the fetus is not fully understood. The cause for differences in leptin concentration between the genders is also controversial.

The present study examined whether sex differences also exist in leptinaemic condition which may provide further clues to the mechanism of sex difference in leptin concentration. Our study reveals significant difference of leptin concentration between male and female fetuses of diabetic mother despite the fact that both male and female babies of DM mother have much higher concentration of leptin than the offspring of non-diabetic mother. In a study, Kostolova *et al* had shown no gender difference in leptin concentration of the offspring of DM mother. Our present study has also shown gender differences of leptin concentration in the offspring of DM mothers. The differences of findings in the gender difference of diabetic offspring of Kostolova and in the DM mother of the present study could be due to differences in body weight between male and female. This study emphasizes that adiposity of the fetus is one of the most determinant factors of leptin concentration.

Although the adiposity cannot be solely responsible for leptin production in the fetus as there is marked difference of leptin concentration between offspring of diabetic and nondiabetic mothers which cannot be fully attributed due to weight differences of the offspring between the groups.

This difference in leptin concentration between offspring of diabetic and nondiabetic mothers group cannot be explained by the presence of higher insulin that exists in the offspring of the diabetic mother because the present study failed to show any correlation between C-peptide and leptin concentration in the offspring of DM mother.[21–22]

Our study is consistent with other studies; our observations may imply that placental leptin might be one of the responsible factors for higher concentration of leptin in the offspring of diabetic mother.

The diabetic male fetus had higher mean value of leptin than that of female which implies that if the female offspring of diabetic mother had similar leptin concentration like that of the male offspring, it could adequately inhibit the hypothalamic control loop.

This negates the higher hypothalamic threshold in leptin adiposity control loop as a possible cause for gender difference. So it seems to be more rational to attribute the differences in leptin production to genetic factors.

## Conclusion

Our study provides information about differences in leptin concentration between two genders in a different leptinaemic situation. This will help to explore the issue further in explaining gender differences in leptin production.

## References

[1] Zhang Y, Proenca R, Maffei M, Barone M, Leopold L, Friedman JM (1994). Positional cloning of the mouse obese gene and its human homologue. Nature.

[2] Pelley Mounter MA, Cullen MJ, Baker MB, Hecht R, Winters D, Boone T (1995). Effects of the obese gene product on body weight regulation in OB/OB mice. Science.

[3] Compfield LA, Smith FJ, Guisez Y, Devos R, Burn P (1995). Recombinant mouse OB protein: Evidence for a peripheral signal linking adiposity and central neural networks. Science.

[4] Considine RV, Sinha MK, Heiman ML, Krianciunas A, Stephens TW, Nyce MR (1996). Serum immuno reactive: Leptin concentration in normal weight and obese humans. N Engl J Med.

[5] Lonnqvist F (1996). The obese gene (OB) and human obesity. News Physiol Sei.

[6] Rosenbaum M, Nicolson M, Hirsch J, Heymsfield SB, Gallagher D, Chu F (1996). Effects of gender, body composition and menopause on plasma concentrations of Leptin. J Clin Endocrinol Metab.

[7] Havel PJ, Kasim-Kara Kas S, Dubuc GR, Mucller W, Phinney SD (1996). Gender difference in plasma leptin concentrations. Nat Med.

[8] Garcia RV, Andrade MA, Rios M, Lage M, Diegueze, Casanueva FF (1997). Serum Leptin levels in normal childern: Relationship to age, gender, body mass index: Pituitary gonadal hormones and pubertal stage. J Clin Endocrinol Metab.

[9] Considine RV, Sinha MK, Heiman ML, Kriauciunas A, Stephens TW, Nyce MR (1996). Serum immunoreactive Leptin concentrations in normal-weight and obese humans. N Engl J Med.

[10] Hassink SG, Sheslow DV, de Lancy E, Opentanova I, Considine RV, Caro JF (1996). Serum leptin in children with obesity: Relationship to gender and development. Pediatrics.

[11] Tome MA, Lage M, Camiña JP, Garcia-Mayor RV, Dieguez C, Casanueva FF (1997). Sex based differences in serum leptin concentrations from umbilical cord blood at delivery. Eur J Endocrinol.

[12] Matsuda J, Yokota I, Iida M, Murakami T, Naito E, Ito M (1997). Serum leptin concentration in cord blood: Relationship to birth weight and gender. J Clin Endocrinol Metab.

[13] Ostlund RE, Yang JW, Klein S, Gingerich R (1996). Relation between plasma leptin concentration and body fat, gender, diet, age and metabolic covariates. J Clin Endocrinol Metab.

[14] Rosenbaum M, Nicolson M, Hirsch J, Heymsfield SB, Gallagher D, Chu F (1996). Effects of gender, body composition, and menopause on plasma concentrations of leptin. J Clin Endocrinol Metab.

[15] Havel PJ, Kasim-Karakas S, Dubuc GR, Mueller W, Phinney SD (1996). Gender differences in plasma leptin concentrations. Nat Metab.

[16] Murakami T, Iida M, Shima K (1995). Dexamethasone regulates obese expression in isolated rat adipocytes. Biochem Biophys Res Commun.

[17] Kennedy A, Gettys TW, Watson P, Wallace P, Ganaway E, Pan Q (1997). The Metabolic Significance of leptin in humans: Gender based differences in relationship toadiposity, Insulin sensitivity and energy expenditure. J Clin Endocr Metab.

[18] Matsuda J, Yokota I, Iida M, Murakami T, Natio E, Ito M (1997). Serum leptin concentration in cord blood: Relationship to birth weight and Gender. J Clin Endocrinol Metab.

[19] Lepercq J, Cauzac M, Lahlou N, Timsit J, Girard J, Auwerx J (1998). Over expression of placental leptin in diabetic pregnancy: A critical role for insulin. Diabetes.

[20] Kostalova L, Lesková L, Kapellerová A, Strbák V (2001). Body mass, plasma leptin, glucose, insulin and C-peptide in offspring of diabetic and non-diabetic mothers. Eur J Endocrinol.

[21] Echwald SM, Clausen JO, Hansen T, Urhammer SA, Hansen L, Dinesen B (1999). Analysis of the relationship between fasting serum leptin levels and estimates of beta-cell function and insulin sensitivity in a population sample of 380 healthy young. Caucasians.

[22] Fehmann HC, Berghofer P, Brandhorst D, Brandhorst H, Hering B, Bretzel RG (1997). Leptin inhibition of insulin secretion from isolated human islets. Acta Diabetologia.

